# A Case Report on an Extremely Rare Disease: Factor XI Deficiency

**DOI:** 10.7759/cureus.10746

**Published:** 2020-10-01

**Authors:** Shobha Mandal, Sumit Gami, Surendra Shah

**Affiliations:** 1 Internal Medicine, Guthrie Robert Packer Hospital, Sayre, USA; 2 Medicine, Universal College of Medical Sciences, Bhairahawa, NPL; 3 Internal Medicine, Nidan Hospital, Kathmandu, NPL; 4 Hematology and Oncology, Guthrie Robert Packer Hospital, Sayre, USA

**Keywords:** factor xi deficiency, fresh frozen plasma, ffp

## Abstract

Factor XI deficiency is a rare condition with an estimated prevalence of about one in one million and is more commonly seen in Ashkenazi Jews (8-9%) due to consanguinity. It occurs because of mutations in the factor XI gene (F11) on chromosome 4(4q35). Patients with this disorder may remain asymptomatic until they undergo any surgical procedure or delivery. The most common sites of bleeding include the oral cavity, pharynx, and genitourinary tract, where there is high fibrinolytic activity. Our patient was asymptomatic his whole life. He never had spontaneous bleeding or bruising; however, he had severe bleeding requiring multiple transfusions of fresh frozen plasma during and after surgeries.

## Introduction

Inherited factor XI (FXI) deficiency is also known as hemophilia C or Rosenthal syndrome. It is an autosomal recessive bleeding disorder that was first described in 1953 in patients who had severe bleeding after dental extractions [1]. It is a relatively rare disease with an estimated prevalence of about one in one million. It is seen more in Ashkenazi Jews (8-9%) due to consanguinity [2]. In the patient population of Ashkenazi Jews, nearly one in eight individuals is found to have heterozygous FXI deficiency. This disorder occurs because of mutations in the FXI gene (F11) on chromosome 4(4q35) [3], and nearly >190 causative mutations have been identified throughout the F11 gene [2,3]. Patients with the homozygous or compound heterozygous disease show a more serious clinical course, whereas those with the heterozygous disease have milder clinical symptoms [4]. Two types of mutations on FXI locus are found to cause FXI deficiency. The type III mutation (Phe283Leu) is found almost solely in Ashkenazi Jews, whereas the type II mutation (Glu117Strop) is also in Iraqi Jews and Arabs [5].

## Case presentation

A 79-year-old gentleman with a past medical history of non-ischemic cardiomyopathy with an ejection fraction of 30-35%, previous automatic implantable cardioverter defibrillator placement, and paroxysmal atrial tachycardia, who was also a BRCA1 carrier was diagnosed with FXI deficiency 30 years ago while undergoing knee replacement surgery. 

Thirty years ago, he had chronic left knee pain and was seen by an orthopedic doctor. He was treated with intra-articular injection with glucosamine and chondroitin injection for his osteoarthritis. Despite treatment with multiple injections, he continued to have pain. He underwent aspiration of the synovial fluid for analysis but the aspirate contained 100 mL of blood. As the patient had no improvement in his symptoms, he underwent left knee replacement surgery. He had massive knee bleeding after the surgery and detailed laboratory work, which showed a prothrombin time of 55 seconds (normal: 26-35.3 seconds). On further workup, he was found to have FXI deficiency with a FXI activity of 35% (normal: 60-150%). Other laboratory values, including prothrombin time, international normalized ratio (1.1 [normal: 0.8-1.2]) and PT% (85% [normal: 80-120%]) were within normal limits. He was diagnosed with heterozygous FXI deficiency. He was treated with 14 units of fresh frozen plasma (FFP) and was discharged home once bleeding stabilized. He followed up with a hematologist and did not have any bleeding problems for years.

**Figure 1:**
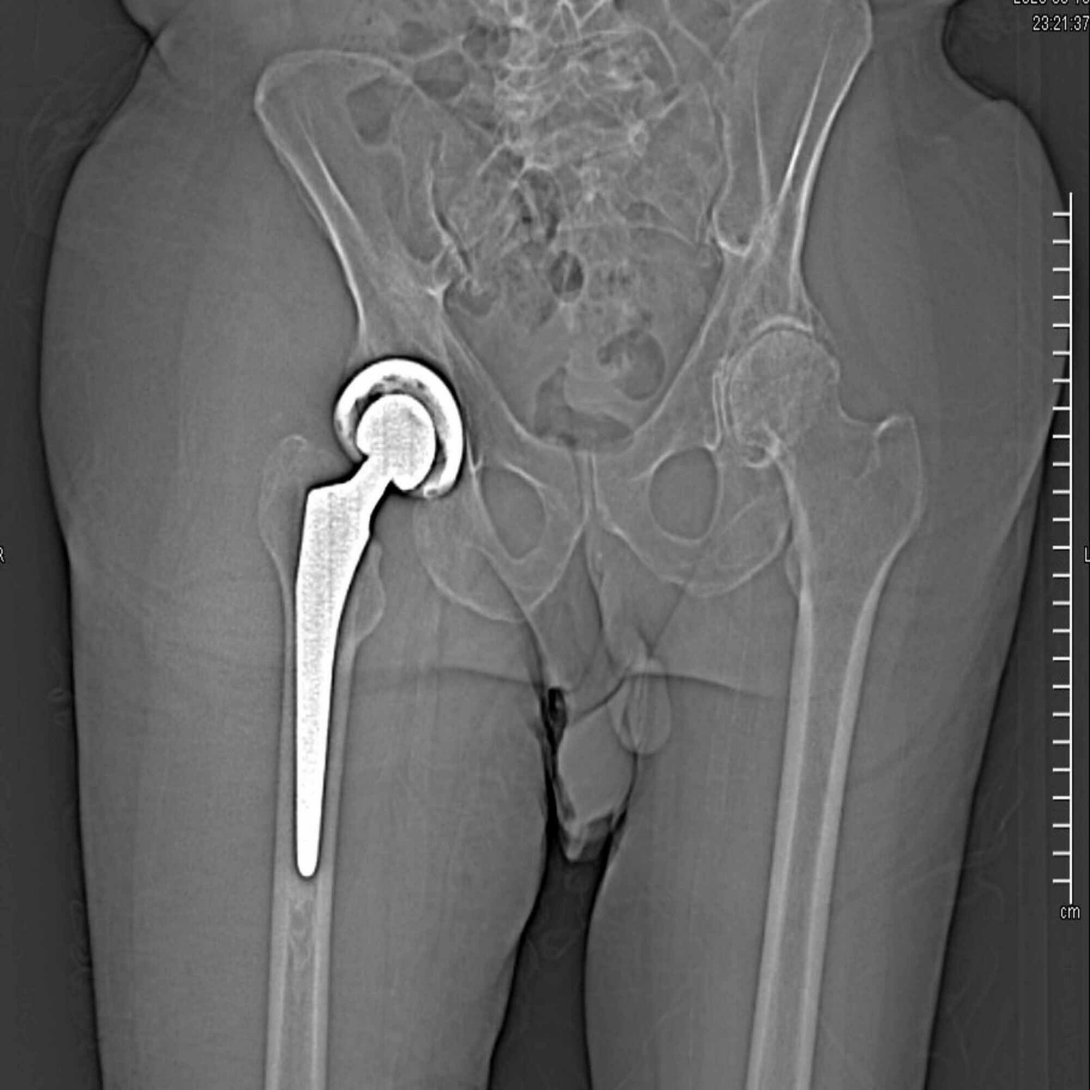
CT showing right hip hematoma extending to the thigh

He presented to our facility with a complaint of right hip pain and was found to have severe osteoarthritis of his right hip. He was scheduled for right hip replacement surgery. The preoperative laboratory work was within normal limits with hemoglobin of 15 mg/dL, activated partial thromboplastin time (aPTT) of 33.4 seconds (normal: 21.3-35.9 seconds). He underwent right hip replacement with a total blood loss of 500 mL. On a postoperative day 1, the patient developed right anterior thigh hematoma. Lab work showed hemoglobin of 12 mg/dL, partial thromboplastin time of 33 seconds (normal: 21.3-35.9 seconds), prothrombin time of 14.4 seconds (normal: 12.0-14.5 seconds). Computed tomography (CT) of the hip showed right hip hematoma extending to the thigh (Figure 1), which was thought most likely secondary to surgical intervention and less likely fasciitis as the patient has no fever or any other symptoms. The patient continued to drop hemoglobin and received multiple transfusions of packed red blood cells. On further workup, FXI activity was 40% (normal: 65-150 %). He was treated with multiple units of FFP for the next nine days. He received four units of FFP on day 1, four units on day 2, three units on day 3, six units on day 4, four units on day 5, seven units on day 6, five units on day 7, two units on day 8, and one unit on day 9. He reported improvement in his swelling overtime and was able to move his leg better compared to earlier stages. A repeat CT of the right lower extremity showed improving right thigh intramuscular hematomas with resolving postoperative changes. CT of the abdomen and pelvis showed no acute findings within the abdomen or pelvis. The patient denied any active bleeding. The hematoma of the right hip resolved, and hemoglobin was stable; hence, he was discharged home on oxycodone, miralax, and physical therapy. Follow-up was scheduled with a hematologist in weeks.

## Discussion

FXI is a plasma glycoprotein that is synthesized in the liver. FXI does not have a major role in initiating coagulation cascade, but it plays a major role in the maintenance of coagulation [6]. FXI is activated by factor XIIa on negatively charged surfaces, known as contact activation. FXI also helps in the regulation of thrombin generation and acts as both procoagulant and antifibrinolytic [7].

Patients with FXI deficiency remain asymptomatic throughout their life until they undergo major surgery, dental extraction, or delivery. Severe spontaneous bleeding is rare, but patients are found to have menorrhagia, epistaxis, increased postpartum hemorrhage, and joint bleeding in individuals involved with sports [8]. Bleeding most commonly occurs in the oral cavity, pharynx, and genitourinary tract, which are areas of high fibrinolytic activity [9]. Patients with hemophilia A and B are found to have hemarthrosis and intramuscular bleeding, which is not seen in FXI deficiency.

In every patient who presents with severe bleeding after any procedure, one should keep FXI deficiency in the differentials and every patient must undergo detailed workup for clotting abnormalities such as platelet count, bleeding time, platelet function tests, prothrombin time, and aPTT. In patients with this deficiency, aPTT can be normal or increased and hence should be further worked up for factor deficiencies including FXI assay [10]. Patients with FXI deficiency are classified as mild (> 40%), intermediate (20-40%), and severe (<15-20% of normal) based on the levels of FXI [11]. The normal blood level of FXI is 70-150 U/dL. There is no evidence showing the linear relation between the factor level and the severity of bleeding, and those patients who are homozygous or compound heterozygous are found to have a factor level of less than 20 U/dL, and those who are heterozygous are found to have a partial deficiency with a factor level of 30-60 U/dL [11].

Patients with FXI are known to bleed excessively after procedures; therefore, anyone who is known FXI deficient should be carefully planned for surgery and should have normal prothrombin time (PT) and platelet count prior to surgery [12]. Global coagulation assays such as thromboelastography/thromboelastometry and thrombin generation testing can be done for the evaluation of phenotypic bleeding and to monitor the response to treatment [13].

FFP is the main approved treatment for FXI deficiency during surgery or with large amounts of bleeding [14]. In patients with severe deficiency, FFP may be inadequate to correct the plasma factor levels. In 90% of the patients, FXI concentrate replacement showed adequate correction of deficiency, but 10% of patients can have increased risk of thrombosis [15]. Monitoring of FXI activity and replacement of factor concentrate or FFP should be conducted as needed [16]. Patients need multiple transfusions to achieve adequate coagulation and are at risk of complications related to volume overloads, such as pulmonary edema, heart failure, and allergic reactions [17]. Any patient with complications of volume overload can be treated with therapeutic plasma exchange [18]. Antifibrinolytic, such as ε-aminocaproic acid or tranexamic acid, fibrin glue, recombinant factor VIIa or factor XIII, and desmopressin can also be used in the treatment to minimize bleeding during surgery [19].

## Conclusions

Hemophilia C is also known as FXI deficiency and is one of the rare disorders causing massive bleeding following procedure and can be life-threatening if not diagnosed and treated in a timely manner. Hence, for any patient with massive bleeding secondary to trauma or procedures, this disorder should be kept in the differentials and managed accordingly.
